# Vitronectin binding protein, BOM1093, confers serum resistance on *Borrelia miyamotoi*

**DOI:** 10.1038/s41598-021-85069-w

**Published:** 2021-03-09

**Authors:** Kozue Sato, Yumi Kumagai, Tsuyoshi Sekizuka, Makoto Kuroda, Tetsuya Hayashi, Ai Takano, Gaowa, Kyle R. Taylor, Makoto Ohnishi, Hiroki Kawabata

**Affiliations:** 1 Department of Bacteriology-I, National Institute of Infectious Disease, Toyama 1-23-1, Shinjuku, Tokyo 162-8640 Japan; 2 Department of Host Defense and Biochemical Research, School of Medicine, Juntendo University, Tokyo, 113-8421 Japan; 3 Pathogen Genomics Center, National Institute of Infectious Disease, Tokyo, 162-8640 Japan; 4 Department of Bacteriology, Faculty of Medical Sciences, Kyushu University, Fukuoka, 819-0395 Japan; 5 Laboratory of Veterinary Epidemiology, Joint Faculty of Veterinary Medicine, Yamaguchi University, Yamaguchi, 753-8511 Japan; 6 Inner Mongolia Key Laboratory of Tick-Borne Zoonotic Infectious Disease, Department of Medicine, College of Hetao, Bayannur, China; 7 College of Veterinary Medicine, Washington State University, Pullman, USA

**Keywords:** Bacterial infection, Immune evasion, Pathogens

## Abstract

*Borrelia miyamotoi*, a member of the tick-borne relapsing fever spirochetes, shows a serum-resistant phenotype in vitro. This ability of *B. miyamotoi* may contribute to bacterial evasion of the host innate immune system. To investigate the molecular mechanism of serum-resistance, we constructed a membrane protein-encoding gene library of *B. miyamotoi* using *Borrelia garinii* strain HT59G, which shows a transformable and serum-susceptible phenotype. By screening the library, we found that *bom1093* and *bom1515* of *B. miyamotoi* provided a serum-resistant phenotype to the recipient *B. garinii*. These *B. miyamotoi* genes are predicted to encode P35-like antigen genes and are conserved among relapsing fever borreliae. Functional analysis revealed that BOM1093 bound to serum vitronectin and that the C-terminal region of BOM1093 was involved in the vitronectin-binding property. Importantly, the *B. garinii* transformant was not serum-resistant when the C terminus-truncated BOM1093 was expressed. We also observed that the depletion of vitronectin from human serum enhances the bactericidal activity of BOM1093 expressing *B. garinii*, and the survival rate of BOM1093 expressing *B. garinii* in vitronectin-depleted serum is enhanced by the addition of purified vitronectin. Our data suggests that *B. miyamotoi* utilize BOM1093-mediated binding to vitronectin as a mechanism of serum resistance.

## Introduction

*Borrelia miyamotoi* was first discovered in Hokkaido, Japan in 1995^1^. Although *B. miyamotoi* is classified to relapsing fever (RF) borreliae, it was discovered in the hard-bodied tick, *Ixodes persulcatus*^1^. *B. miyamotoi* has also been detected from *Ixodes scapularis* and *Ixodes pacificus* in North America^2–4^ and *Ixodes ricinus* in Europe^5,6^. The first cases of *B. miyamotoi* infection in humans were reported in Russia and were referred to as “Emerging RF”^7^. Following the initial report, several cases of *B. miyamotoi* infection have been confirmed in humans in the United States, Europe, and Asia^8–13^.

Emerging RF (recently renamed *B. miyamotoi* disease, or BMD) is a systemic illness causing fever, headache, myalgia, arthralgia, elevated liver enzymes, neutropenia, and thrombocytopenia^7,14^, and several cases of meningitis have been reported^8–10,15^. Spirochetemia has been reported in cases of BMD, and survival of spirochetes in the bloodstream may be important in establishing systemic infection. Resistance to human complement was demonstrated for *B. miyamotoi* in 2014^16^, and the complement binding and inhibitory protein A (CbiA) has been identified as a serum-resistance factor in *B. miyamotoi*^17^. However, the mechanisms utilized by the BMD pathogen, *B. miyamotoi*, are not fully understood.

Although genetic approaches such as mutagenesis and complementation have been employed to study genes of genus *Borrelia* over the last few decades, these processes have not been established for *B. miyamotoi*. Röttgerding et al., however, successfully identified and characterized the contribution of CbiA to serum resistance of *B. miyamotoi *in vitro using a surrogate strain, *B. garinii* G1^17^. We, therefore, employed a similar surrogate system by first establishing a transformable/serum susceptible *Borrelia* strain to use in our investigation. Using this strain, we attempted to comprehensively screen genes involved in serum resistance of *B. miyamotoi* and found that a vitronectin (Vn)-binding protein contributed to serum resistance of *B. miyamotoi *in vitro. Vn is a serum glycoprotein that circulates in the bloodstream and has roles in many biological processes including the regulation of the terminal pathway of complement in which it inhibits the C_5b7_ complex formation and C_9_ polymerization^18,19^. Our data suggests *B. miyamotoi* may utilize Vn-binding to evade the complement system in human serum.

## Results

### Identification of serum-sensitive *B. garinii* HT59G which shows a transformable phenotype

**Figure 1:**
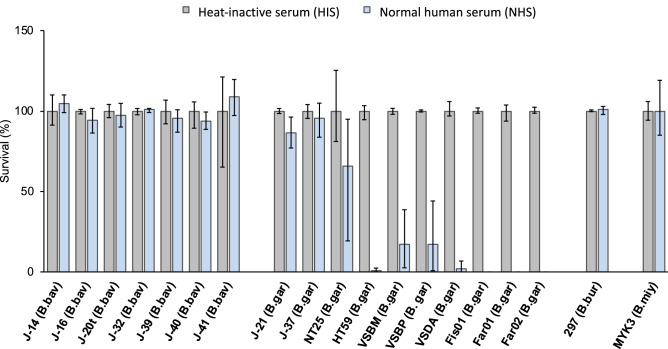
Serum susceptibility of *Borrelia* strains used in this study. Spirochetes were incubated in either 40% normal human serum (NHS) or heat-inactivated serum (HIS) for 5 days at 37 °C. Cell viability was assessed using microscopic counts of cells in 10 fields under ×300 magnification. The figure depicts the means, and error bars represent the positive and negative errors of the mean of triplicates from one representative experiment. *Borrelia* species names abbreviated as follows: *Borrelia bavariensis* (B. bav), *B. garinii* (B. gar), *B. burgdorferi*, (B. bur), and *B. miyamotoi* (B. miy).

We first sought to evaluate the susceptibility of *Borrelia* strains to human serum in detail using strains isolated from different biological and geographical samples. For this purpose, 17 *Borrelia* strains of *B. garinii* and *B. bavariensis* were examined for serum-sensitivity by determining the survival rate following treatment with 40% Normal human serum (NHS) or Heat-inactivated human serum (HIS) for 16 h (Figure 1). Of these 17 strains, nine strains (*B. bavariensis* strains J-14, J-16, J-20t, J-32, J-39, J-40, J-41 and *B. garinii* strains J-21, J-37) obtained from the skin of Lyme disease patients, two *B. garinii* strains (strains VSBM and VSBP) isolated from cerebrospinal fluid (CSF) of patients, and one *B. garinii* (strain NT25) isolated from a tick exhibited a serum-resistant phenotype. One *B. garinii* strain (strain VSDA) isolated from patient CSF and four strains of *B. garinii* (strains Fis01, Far01, Far02, and HT59) isolated from ticks were serum-sensitive. These serum-sensitive strains were selected as candidate hosts for gene library construction of *B. miyamotoi*. To investigate the transformability of these *B. garinii* strains, the shuttle vector pBSV2 was electroporated into each serum-susceptible *B. garinii* strain. Among the five strains tested, transformants were obtained only from *B. garinii* strain HT59. We therefore picked 10 single colonies of strain HT59 and established 10 clones. Of these 10 clones, clone G also showed a transformable phenotype. When *B. garinii* strain HT59G was transformed with plasmid pBSV2, an average of 15 transformants was obtained per 1 µg of plasmid DNA (Table 1).

**Table 1:** Efficiency of transformation of human serum sensitive-*B. garinii* strains with pBSV2.

Strain	Number of kanamycin-resistant colonies /µg of DNA			
	Exp.1	Exp.2	Exp.3	Mean
VS DA	ND	NT	NT	–
Fis01	ND	NT	NT	–
Far01	ND	NT	NT	–
Far02	ND	NT	NT	–
HT59 clone G	19	19	7	15

*ND* not detected, *NT* not tested.

### Construction of plasmid archives for *B. garinii* HT59G transformation

At the time of this study, the genome of *B. miyamotoi* strain MYK3 was not available. Therefore, candidate genes encoding membrane proteins were selected from the genome sequence of *B. miyamotoi* strain FR64b, which is published in GenBank (Acc. Nos. CP004218–CP004266). From this database, 649 open reading frames (ORFs) that were predicted to be non-chromosomal encoding were extracted. Of these 649 ORFs, 90 ORFs were predicted to be displayed on the bacterial surface of *B. miyamotoi* using SignalP or LipoP analysis. For each of these 90 ORFs, specific PCR primers were used for DNA amplification. All ORF PCR products were detected from template genomic DNA of *B. miyamotoi* strain MYK3. The shuttle vector was created for the 90 ORFs by combining linearized pBSV2 and each PCR fragment using the In-Fusion procedure according to the manufacturer’s instructions (see “Materials and methods”****). Of these 90 ORFs, 84 ORFs were isolated from *E. coli* DH5α. Of these 84 ORFs, transformation of *B. garinii* HT59G was successful with 76 ORFs (coverage ratio: 84.4%).

### *bom1093* and *bom1515* provide serum-resistance phenotype to recipient *Borrelia*

**Figure 2:**
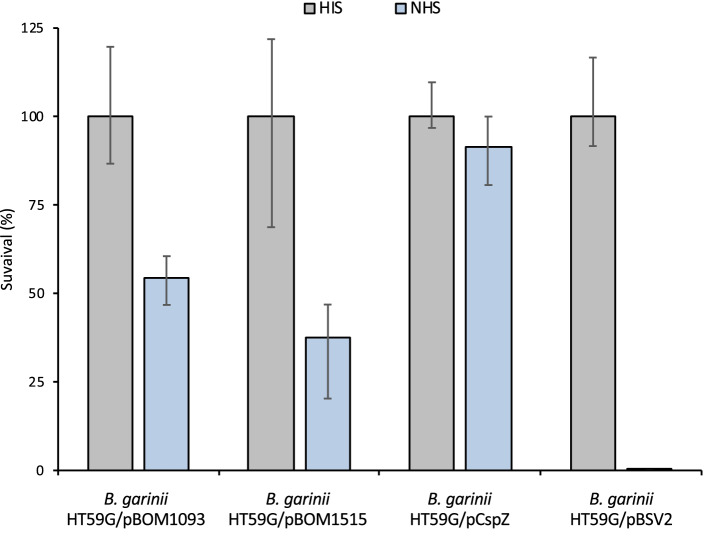
Quantification of serum susceptibility for *Borrelia garinii* transformants. Each transformant was incubated in either 40% NHS or HIS for 16 h at 34 °C. The colony forming unit (CFU) represents the number of colonies formed. The mock strain (*B. garinii* HT59G/pBSV2) and serum-resistant transformant (*B. garinii* HT59G/CspZ) were used as controls. The figure depicts the average CFU, and error bars represent the positive and negative errors of the mean of triplicates. *p value (≤ 0.01) was calculated by Student’s *t* test.

In order to investigate serum resistance of the 76 transformants, we subjected them to a screening test. After culturing for 5 days with 40% NHS, survival of the bacteria was observed using a dark-field microscope. The negative control (*B. garinii* HT59G/pBSV2) was completely destroyed in the presence of NHS. In contrast, *B. garinii* HT59G/pCspZ, with introduced Complement Regulator-Acquiring Surface Protein-2 gene (*cspZ*) of *B. burgdorferi* strain 297, a gene encoding a factor H (FH)-binding protein, used as a survival control, showed growth. Of the 76 transformants examined, two (*B. garinii* HT59G/pBOM1093 and HT59G/pBOM1515) exhibited a serum-resistant phenotype in a bactericidal assay. These transformants were further subjected to a quantitative serum-sensitivity assay. The transformed strains treated with 40% NHS or HIS for up to 16 h were plated on a 1% soft agar overlay on BSK-M plates with kanamycin. The *B. garinii* HT59G/pBSV2 and *B. garinii* HT59G/pCspZ strains were used as controls for serum susceptibility and serum resistance to NHS, respectively. The survival ratio was calculated using colony-forming units (CFUs) of NHS-treated cells and HIS-treated cells. In this study, *B. garinii* HT59G/pCspZ and *B. garinii* HT59G/pBSV2 showed survival ratios of 91% and 0.1%, respectively (Fig. 2). Moreover, *B. garinii* HT59G/pBOM1093 and *B. garinii* HT59G/pBOM1515 showed 54.6% and 37.5% survival ratios, respectively. Amino acid sequences of BOM1093 and BOM1515 from the strain *B. miyamotoi* MYK3 showed high similarity (94% identity) with each other, and the BOM1093 sequence of *B. miyamotoi* strain MYK3 was identical to BOM1093 of the strain FR64b (Acc. No. NZ_CP004225).

### Proteinase K accessibility of BOM1093

**Figure 3:**
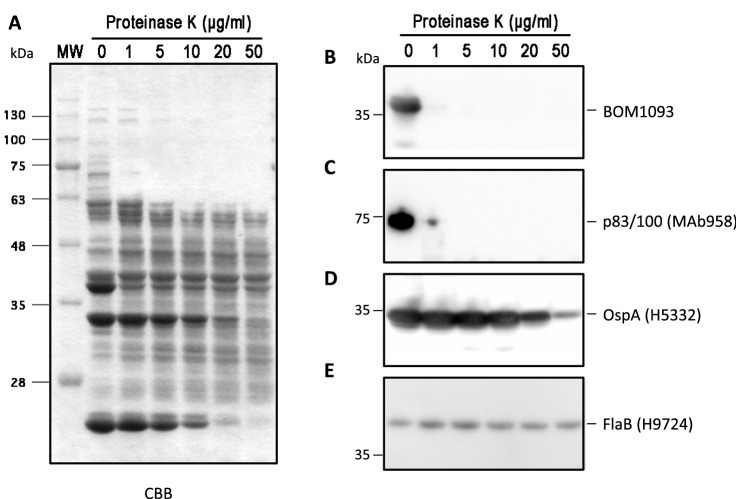
Protease treatment affects surface exposed protein, BOM1093. *B. garinii* HT59G/pBOM1093 cells were incubated with the indicated concentrations of proteinase K. After 1 h of incubation, the cells were lysed by SDS-PAGE buffer, and each protein lysate was separated by 12.5% SDS-PAGE. (**A**) Total proteins were detected by Coomassie Brilliant Blue (CBB) staining, (**B**) BOM1093 was detected with anti-BOM1093 rabbit serum (dilution 1:1000), (**C**) P83/100 was detected with MAb958 (dilution 1:1000), and (**D**) OspA was detected with MAb H5332 (dilution 1:1000). (**E**) Flagellin was detected with MAb H9724 (dilution 1:1000) by western blotting.

In silico analyses (SignalP and LipoP) predicted BOM1093 to be a membrane protein. To further investigate the localization of the BOM1093 protein, a Proteinase K accessibility test was performed. Proteinase K treatment was performed for *B. garinii* HT59G/pBOM1093. As shown in Fig. 3, both P83/100 and OspA were digested by proteinase K treatment in a dose-dependent manner. BOM1093 was also accessible to proteinase K. In contrast, flagellin protein, which reacted with monoclonal antibody H9724, was resistant to proteinase K treatment.

### Human serum Vn co-precipitated with His-tagged BOM1093

**Figure 4:**
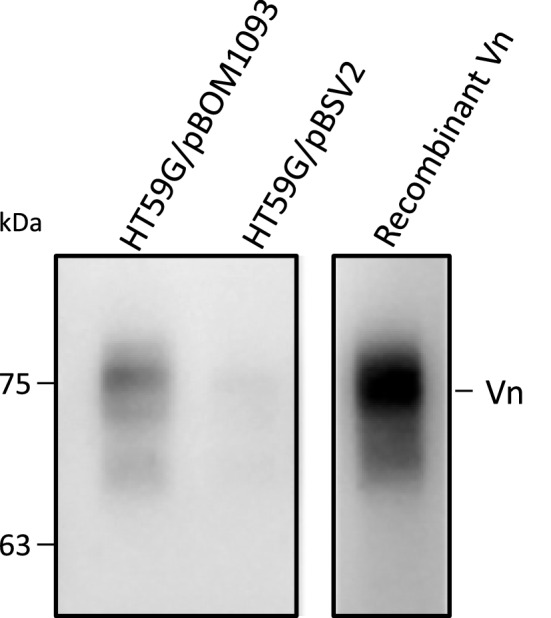
Human serum Vn is co-precipitated with BOM1093. *B. garinii* HT59G expressed 6xHis-tagged BOM1093 (~ 10^8^ cells/tube) were incubated with 20% NHS for 1 h at 37 °C and washed 3 times with Tris–HCl buffer (pH 7.0) containing 5 mM Pefabloc SC. The 6xHis-tagged BOM1093 from sonicated cells was captured using Ni–NTA magnetic beads. The captured proteins were separated on a 10% SDS-PAGE gel, transferred to PVDF membrane, and Vn was detected by western blotting. *B. garinii* HT59G/pBSV2 was used as a Mock control.

Serum resistance phenotype of *Borrelia* is due to its ability to bind complement regulator (for e.g., FH). Therefore, we examined the binding of *B. garinii* HT59G/pBOM1093 to complement regulators FH, factor H-like protein 1 (FHL-1), factor I, properdin, carboxy peptidase N, C4b binding protein (C4BP), C1 inhibitor, complement factor H-related protein 1, clusterin and vitronectin (Vn). *B. garinii* HT59G/pBOM1093 (6xHis-tagged on C terminus) incubated in 20% NHS was tested by a pull-down assay using Ni–NTA magnetic beads. The only complement regulator that bound to BOM1093 was Vn, which is reported to inhibit the terminal pathway of the complement system (e.g. C9 polymerization). In contrast, Vn was not detected when *B. garinii* HT59G/pBSV2 was used as a prey antigen (Fig. 4). Based on this data, we conducted further analyses for Vn and BOM1093.

### *B. garinii* HT59G/pBOM1093 binds purified-recombinant Vn in a dose-dependent manner

**Figure 5:**
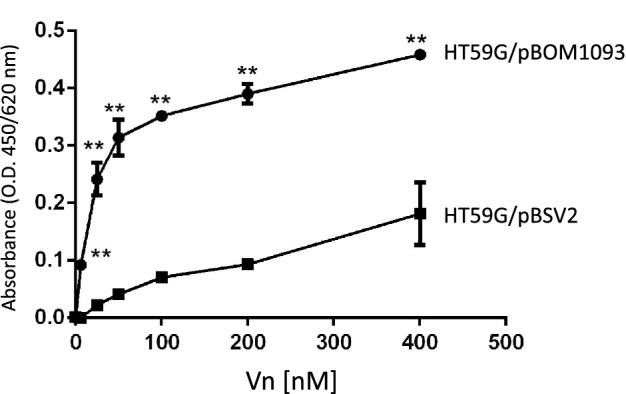
Vn-binding to *B. garinii* transformants using recombinant Vn. Vn-binding to *B. garinii* HT59G/BOM1093 was quantified by ELISA and depicted as the ratio of O.D. 450 nm to the background absorbance at 620 nm. Recombinant human Vn (6.25–400 nM) was used for this study. Values are means ± SD of three independent experiments. Values were compared and analyzed between *B. garinii* HT59G/BOM1093 and *B. garinii* HT59G/BOM1093. **p value (≤ 0.001) was calculated by Student’s *t* test.

To confirm the results of the pull-down assay, we performed an ELISA assay to examine the binding of purified recombinant Vn to *B. garinii* HT59G/pBOM1093. A Vn-binding ELISA assay found dose-dependent and saturation binding of recombinant Vn for *B. garinii* HT59G/pBOM1093. In contrast, Vn binding was not detected when *B. garinii* HT59G/pBSV2 was tested. The results are shown in Fig. 5. The K_D_ value for the interaction was estimated to be 10.8 nM (95% Confidential interval range 8.5–13.0 nM).

### C-terminal region of BOM1093 is predicted to contribute to serum resistance

**Figure 6:**
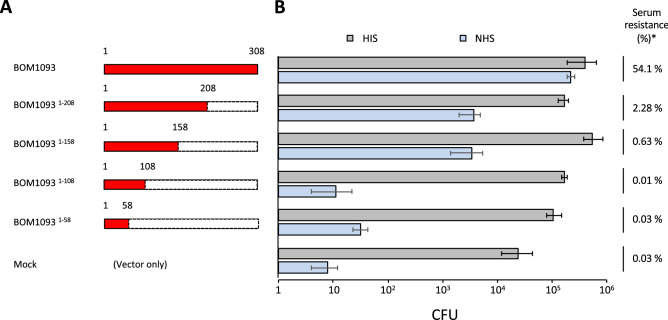
The effect of C-terminal truncation of BOM1093 on serum susceptibility. Schematic picture of C-terminal truncation of BOM1093 (left) and serum susceptibility of each mutant (right) are shown. *Ratio of serum resistance (%) was calculated by dividing the CFUs of NHS by CFUs of HIS, respectively.

Next, we attempted to determine the region of BOM1093 that contributes to serum resistance. Briefly, the effect of truncation of BOM1093 on serum susceptibility was examined. In this study, four truncated mutants of BOM1093 were created by gradually deleting different nucleotide sequences from the *bom1093* gene; BOM1093^1–208^ (*bom1093*∆209–308), BOM1093^1–158^ (*bom1093*∆159–308), BOM1093^1–108^ (*bom1093*∆109–308), and BOM1093^1–58^ (*bom1093*∆59–308) were generated by PCR. These truncated genes were concatenated with *pflaB* on pBSV2 and were then introduced into *B. garinii* HT59G. The four mutants used in this study are schematically represented in Fig. 6A. All four truncated mutants survived equally well in the HIS (Fig. 6B). As previously mentioned, *B. garinii* HT59G/pBOM1093 showed a serum-resistant phenotype with a survival rate of 54%, and the mock control (*B. garinii* HT59G/pBSV2) demonstrated a survival rate of less than 1% (Fig. 6B). All the truncated mutants (*B. garinii* HT59G/pBOM1093^1–208^, HT59G/pBOM1093^1–158^, HT59G/pBOM1093^1–108^, and HT59G/pBOM1093^1–58^) were observed to be serum-susceptible phenotypes (all with a survival ratio less than 2.3%) with dramatically decreased survival compared to *B. garinii* HT59G/pBOM1093. These results suggest that the C-terminal region of BOM1093 between 209 and 308 is important for converting *B. garinii* HT59G/pBOM1093 to a serum-susceptible phenotype.

### C-terminal region of BOM1093 is required for Vn-binding

**Figure 7:**
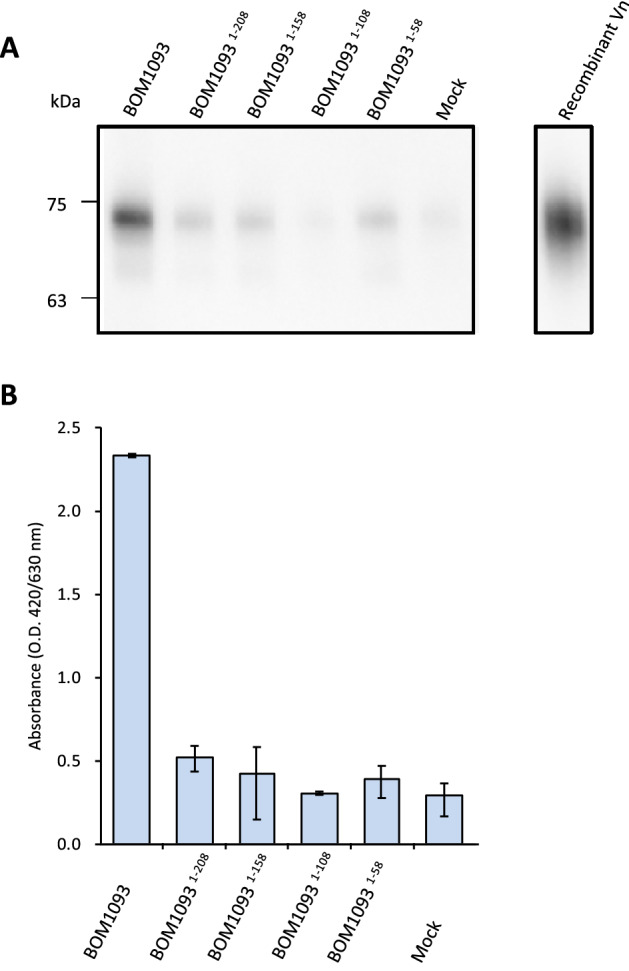
Decrease of serum Vn binding in C-terminal truncation mutants. *B. garinii* expressed BOM1093 or C-terminal truncation mutants were used in this study. *B. garinii* HT59G/pBSV2 was used as Mock control. (**A**) Detection of co-precipitated serum Vn by western blotting. M.W. indicated molecular weight marker. (**B**) The amount of co-precipitated Vn was quantified by ELISA.

To define the Vn-binding facilities of BOM1093, C-terminal-truncation mutants were subjected to a pull-down assay. Although intact 6xHis tagged-BOM1093 co-precipitated with Vn, none of the truncated proteins (6xHis tagged-BOM1093^1–208^, 6xHis tagged-BOM1093^1–158^, 6xHis tagged-BOM1093^1–108^, and 6xHis tagged-BOM1093^1–58^) co-precipitated with Vn (Fig. 7A,B). Moreover, an ELISA assay revealed that *B. garinii* HT59G/pBOM1093^1–208^, in which the C-terminal residues 209–308 were deleted, showed reduced binding compared to *B. garinii* HT59G/pBOM1093 (Fig. 7B). Furthermore, Vn-binding was not detected in the other mutants due to deletion of the C terminus of BOM1093. These results suggest that the 209–308 region of the C-terminal amino acids of the BOM1093 protein is essential in enabling *B. garinii* HT59G/pBOM1093 to bind to Vn in vitro.

### Depletion of Vn enhanced bactericidal activity of serum-resistant *B. garinii* HT59G/pBOM1093

**Figure 8:**
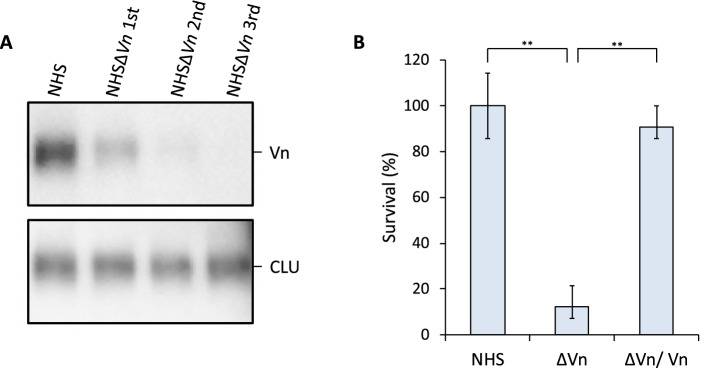
Vn-depleted human serum has bactericidal activity against serum-resistant *B. garinii* HT59G/pBOM1093. (**A**) Serum Vn was depleted for NHS. Vn depletion from human serum was repeated three times (HSΔVn 1st, HSΔVn 2nd, and HSΔVn 3rd). Levels of Vn and clusterin (CLU) in tested sera were determined by western blotting. (**B**) Survival ratio was calculated for *B. garinii* HT59G/pBOM1093. ΔVn indicates the HSΔVn third sample (HSΔVn 3rd). ΔVn/Vn indicates the tested serum, which was supplemented with 1 mM of recombinant Vn to the HSΔVn 3rd. **p ≤ 0.01.

It is well characterized that NHS-derived Vn inhibits the complement system. Therefore, removal of Vn from NHS enhances bactericidal activity. In this study, we prepared human serum depleted of Vn (HSΔVn) and used it in the serum susceptibility assay of *B. garinii* transformants. In this study, Vn depletion was confirmed by western blotting with clusterin (CLU), as a positive control (Fig. 8A). The Vn-depleted serum was subjected to bactericidal assays using *B. garinii* HT59G/pBOM1093 and the mock control (*B. garinii* HT59G/pBSV2). *B. garinii* HT59G/pBOM1093 showed a significant increase in serum susceptibility when incubated with HSΔVn (Fig. 8B). However, when 1 mM of purified recombinant Vn was added to the HSΔVn, serum-resistance was observed for *B. garinii* HT59G/pBOM1093.

## Discussion

In previous studies, several transformable *Borrelia* strains have been used as surrogate strains for serum susceptibility analysis. *B. burgdorferi* strain B313, which is a derivative of strain B31, is one of the transformable and serum susceptible strains^20^. The strain is convenient for genetic analysis because it forms colonies on a semi-solid BSK agar plate. *B. garinii* strains G1 and 50.97 have also been used for genetic analyses of serum susceptibility because these strains are transformable and susceptible to human serum^21,22^. However, transformants of *B. garinii* strains were isolated using a limiting dilution technique in liquid BSK medium. This process for isolating clonal transformants is an intricate procedure that requires several weeks. Furthermore, more than 10 µg of plasmid DNA is required for efficient transformation for these strains. In this study, we established another transformable and serum-susceptible *B. garinii* HT59G. Transformation efficacy of the strain was 15 transformants/µg plasmid DNA when pBSV2 plasmid was used, and transformants were isolated from semi solid agar plates. These results suggest that the *B. garinii* strain HT59G used in our study may be a useful and convenient tool to investigate serum resistance mechanisms of borreliae.

Bacterial pathogens display proteins on their surface, some of which bind　complement regulators and inhibit the host complement system. Several reports have indicated that borreliae have the ability to evade the innate immune system. Lyme disease borreliae produce the protein p43 which binds C4BP to the bacterial surface, thereby regulating the classical and lectin pathways through degradation of C4b^23^. Most Lyme disease borreliae produce several complement regulator-acquiring surface proteins (CRASPs): CRASP-1 to CRASP-5 (CspA, CspZ, and Erps). CspA and CspZ are ligands for FH and/or FHL-1. These proteins can also bind C3b and then promote C3b degradation on the bacterial surface to regulate the complement pathway^24^. Erps (ErpP, ErpC, and ErpA) can also bind to FH and contribute to serum resistance of Lyme disease borreliae; however, the biological significance of these interactions remains unclear. RF borreliae also express complement regulator-binding proteins on their surface^25^. BHA007 (*B. hermsii*) and CihC (*B. recurrentis* and *B. duttonii*) can bind C4BP. CihC is also known as C1-INH-binding protein. The binding of BHA007/CihC to C1-INH and C4BP on the bacterial surface prevents the formation of C1 and mannan/mannose-binding lectin-associated serine protease complexes and induces the cleavage of C4b, respectively, resulting in inhibition of the classical and lectin pathways. FhbA of *B. hermsii*, BpcA of *B. parkeri*, and HcpA of *B. recurrentis* bind FH (and/or FHL-1), which inhibits the alternative pathway through C3b cleavage. BBK32 of Lyme disease borreliae, known for both fibronectin^26^ and glycosaminoglycan binding^27^, was recently reported to be a C1r-binding protein. By binding to the inactive form of C1r, BBK32 blocks the formation of the active C1 complex thereby inhibiting the classical pathway^28^. CspA, BGA66 and BGA71 of Lyme disease borreliae bind C7, C8, and C9, and may inhibit the terminal cascade of the complement pathway^29,30^. A CD59-like protein of *B. burgdorferi* that binds to C9 has also been suspected; however, the borrelial factor was not identified^31^. These interactions contribute to the inhibition of the membrane attack-complex, thereby preventing bacteriolysis. Complement regulation by Vn binding, however, has not been reported in *Borrelia*.

Vn was discovered in 1967^32^ and initially called S-protein, but was later renamed by Hayman et al.^33^. Vn binds to the membrane attachment site of the C5b-7 complex, thereby blocking insertion into the target membrane and inhibiting C9 polymerization and attachment^18,34^. Bacterial pathogens thereby evade bactericidal action by the complement system through binding host Vn to their surfaces^35^. Vn-dependent serum resistance has been well studied for several pathogens. The serum resistance mechanism by Vn binding has been reported in LcpA of *Leptospira* species, Lpd of *Pseudomonas aeruginosa*, PE of non-typable *Haemophilus influenzae*, and UspA2 of *Moraxella catarrhalis*^36–39^. In this study, we revealed that *B. miyamotoi* BOM1093 acts as a virulence factor contributing to human serum resistance by binding to serum Vn.

In previous studies, *B. miyamotoi* has shown a serum-resistant phenotype^16,40^, and only CbiA (locus tag BOM1283, Acc. No. AHH05826) has been identified as a factor responsible for this phenotype^17^. Briefly, it was shown that CbiA binds to FH and interacts to complement components (C3, C3b, C4, C4b, C5, and C9), thereby potentially blocking the alternative, classical, and terminal pathways of the complement system. However, it has not been shown whether this multi-functional protein is also capable of binding Vn. To our knowledge, this is the first study to report that Vn-binding to a borrelial factor promoted serum resistance of *B. miyamotoi*.

BOM1093 was identified as the Vn-binding protein in this study. Using Protein Basic Local Alignment Search Tool (BLASTP) analysis, BOM1093 and BOM1515 proteins were identified as related to antigen P35^41^ in Lyme disease borreliae. In addition, the amino acid residue of BOM1093, which was suggested to be an important region for Vn binding, had a homology of 94.0% identity with the sequence of BOM1515. BOM1515 is also expected to confer serum resistance to *B. miyamotoi* by Vn-binding. From BLASTP analysis, we found that BOM1093 was conserved in RF borreliae including hard-tick-borne RF borreliae. Sequence similarity of BOM1093 ranged from 59 to 100% in *B. miyamotoi* and from 56 to 68% in RF borreliae. The *bom1093* lineage possessed by RF borreliae may have a function similar to that of *B. miyamotoi bom1093*. In this study, we conclude that the *C*-terminal region of BOM1093 is involved in Vn-binding. However, it is also possible that the *C*-terminal region of BOM1093 is required for structural stability of the protein and that its removal results in a protein with disrupted structure and function. To resolve this question, further investigation is required.

## Conclusion

In conclusion, using a newly established transformable *B. garinii* strain, we revealed that *B. miyamotoi* has the ability to bind Vn through the membrane protein BOM1093. We hypothesize that Vn-binding may contribute to pathogenicity of *B. miyamotoi* in humans by allowing it to evade the serum complement system. This is the first study to report that Vn-binding is associated with serum resistance of *Borrelia*.

## Materials and methods

### Bacterial strains and culture conditions

Borrelial strains used in this study are listed in Table 2. *B. garinii* strains J-21, J-37, VSBM, VSBP, VSDA, Fis01, Far01, Far02, HT59, NT25, *Borrelia bavariensis* strains J-14, J-16, J-20t, J-32, J-39, J-40, J-41, *Borrelia burgdorferi* 297, and *B. miyamotoi* MYK3 were used in this study. *B. garinii* HT59G was isolated from strain HT59 by subsurface colony formation^42^. These *Borrelia* strains were grown at 34 °C in Barbour–Stoenner–Kelly (BSK)-M medium^43^ supplemented with 7% rabbit serum with or without selectable antibiotics. For antibiotic selection of shuttle vector transformants, kanamycin (200 µg/ml) was used. *Escherichia coli* DH5α was used for the preparation of plasmids for electroporation into *B. garinii* HT59G.

**Table 2:** *Borrelia* strains used in this study.

*Borrelia* species	Strain	Isolated source	References
*Borrelia garinii*	J-21	Human skin biopsy, Japan	^44^
	J-37	Human skin biopsy, Japan	^44^
	NT25	*Ixodes persulcatus*, Japan	^44^
	HT59	*I. persulcatus*, Japan	^45^
	HT59G	Daughter strain cloned from the parent strain HT59	This study
	VSBM	Human cerebrospinal fluid, Switzerland	^46^
	VSBP	Human cerebrospinal fluid, Switzerland	^46^
	VSDA	Human cerebrospinal fluid, Switzerland	^46^
	Fis01	*Ixodes uriae*, Denmark	^47^
	Far01	*I. uriae*, Denmark	^47^
	Far02	*I. uriae*, Denmark	^47^
*Borrelia bavariensis*	J-14	Human skin biopsy, Japan	^44^
	J-16	Human skin biopsy, Japan	^44^
	J-20t	Tick, fed on human skin, Japan	^44^
	J-32	Human skin biopsy, Japan	^44^
	J-39	Human skin biopsy, Japan	^44^
	J-40	Human skin biopsy, Japan	^44^
	J-41	Human skin biopsy, Japan	^44^
*Borrelia burgdorferi*	297	Human cerebrospinal fluid, United States	^48^
*Borrelia miyamotoi*	MYK3	*I. persulcatus*, Japan	^43^

### Preparation of NHS and HIS

NHS from a human blood donor without history of a borrelial infection was used. The serum was confirmed serologically negative by the absence of IgG and IgM antibodies against *Borrelia* spp. A serum diagnostic test was performed for Lyme disease by immunoblotting using a commercial kit, *recom*Line Borrelia IgM/IgG (Mikrogen GmbH, Neuried, Germany). We obtained ethical approval for the use of human serum (The details are provided in the Medical Ethics section). HIS was prepared by incubating NHS at 56 °C for 30 min.

### Screening assay for serum sensitivity

The serum sensitivity of each borrelial strain was assessed using cells harvested from mid-log phase cultures. The cells (~ 10^7^ cells/ml) were incubated in 40% NHS or HIS at 37 °C. After incubation for 16 h, cell viability was assessed using dark-field microscopic counts of moving cells in 10 fields under 300× magnification. Data are presented as percent survival, calculated as follows: (average number of moving cells/numbers of morphologically collapsed cells per ten 300× magnification fields) × 100. In each assay, the *B. burgdorferi* strain 297 and *B. garinii* VSDA were used as survival control and serum-sensitive control, respectively^35,49^.

### Electroporation of *Borrelia* strains

Electroporation of serum-sensitive *Borrelia* strains was performed as described previously^50,51^ with minor modifications. Briefly, *Borrelia* strains were grown in BSK-M medium and harvested at the late-log phase (5 × 10^7^–1 × 10^8^ cells/ml), which were subsequently prepared for electroporation by washing once with phosphate-buffered saline (PBS) (FUJIFILM Wako Pure Chemical Corp., Osaka, Japan) and twice with electroporation solution [EPS: 0.27 M sucrose, 10% [v/v] glycerol] by centrifugation at 5000×*g* for 15 min at 4 °C. Freshly prepared competent cells (approximately 10^9^ cells in 50 µl of electroporation solution) were transformed with 0.3–2.0 µg of plasmid DNA. After electroporation at 2.5 kV, 25 μF, and 200 Ω, bacterial cells were immediately resuspended in 1 ml of prewarmed BSK-M medium and incubated for 20–24 h at 34 °C. The cultures were then plated as a 1% soft agar overlay^42^ on BSK-M plates with kanamycin (200 µg/ml) and incubated for 2 weeks at 34 °C. To determine transformation efficiency (transformants/µg DNA), plasmid pBSV2 was used^52^. The colonies were picked up from the BSK-M plates and were cultured until mid-log phase. The presence of the kanamycin resistance gene (*kanR*) of post-transformation *Borrelia* strains was confirmed by *kanR-*PCR using the primers mentioned in the Supplemental Table S1.

### In silico analysis of ORFs

The plasmid sequences of *B. miyamotoi* strain FR64b (Acc. Nos. CP004218–CP004266) were subjected to in silico analyses. From these sequences, 649 ORFs (locus-tag nos. BOM 0875–BOM 1523) were extracted and translated into amino acid sequences. These 649 polypeptides were analyzed using SignalP 4.1 (http://www.cbs.dtu.dk/services/SignalP/)^53^ and LipoP 1.0 (http://www.cbs.dtu.dk/services/LipoP/)^54^ to predict the existence and location of signal peptide cleavage sites, and to predict the existence of lipoprotein signal peptides, respectively.

### Plasmid construction and transformation of recipient *B. garinii*

**Figure 9:**
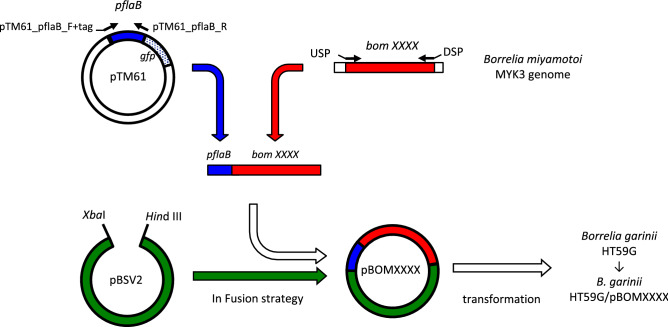
Schematic model of plasmid construction and transformation of recipient *Borrelia.* These were abbreviated in this figure as follows; promoter region of *flaB* gene (*pflaB*), upstream 5′-DNA primer (USP), and downstream 3′-DNA primer (DSP), respectively.

The outline of plasmid construction is summarized in Fig. 9. The shuttle vector pBSV2 was used for transformation of *B. garinii* HT59G. Plasmid pBSV2 was originally constructed by Stewart et al.^52^. The flagellin gene (*flaB*) promoter (p*flaB*) was used for gene expression of the borrelial gene in the recipient *Borrelia* strain. DNA fragment of p*flaB* on plasmid pTM61^55^ was amplified by PCR using a set of primers: pTM61_pflaB_R and pTM61_pflaB_F + tag (Supplemental Table S1). Plasmids for borrelial transformation were constructed using the In-Fusion HD Cloning System (Clontech Laboratories, Mountain View, CA, USA). Briefly, plasmid pBSV2 was linearized by digestion with restriction enzymes (Hind III and Xba I, Takara Bio, Shiga, Japan). DNA fragments of each ORF of *B. miyamotoi* were amplified by PCR using specific primers for each ORF. Concatenation of p*flaB*, each amplified fragment from the genomic DNA of *B. miyamotoi* strain MYK3 and linearized pBSV2 was performed using the In-Fusion system according to manufacturer’s instructions. The primers used for amplification of DNA fragments from *B. miyamotoi* are listed in the Supplemental Table S2. The constructed plasmid was propagated using *E. coli* DH5α and purified with the Qiaprep Spin Miniprep kit (QIAGEN, Calif, USA). The purified plasmids were subjected to nucleotide sequencing to ensure no mutations introduced during the cloning process. The oligonucleotide primer pair (T7 and AS-T, Supplemental Table S1), which amplified the DNA fragment of multi-cloning site of pBSV2, was used for PCR amplification and sequencing. Each plasmid was used to transform *B. garinii* HT59G. The transformed *B. garinii* HT59G was picked up from the BSK-M plate containing 200 µg/ml kanamycin. Transformation with plasmid was confirmed by *kanR*-PCR and PCR using DNA primers T7 and AS-T.

### Serum resistance assay of transformed* B. garinii* HT59G clones

For initial screening, each *B. garinii* HT59G transformant (~ 10^7^ cells/ml) was incubated with rabbit serum-free BSK-M medium with 40% NHS for 5 days at 34 °C, and the survival of transformants was assessed using dark-field microscopy. For calculation of serum resistance, the transformants (~ 10^6^ cells/ml), which showed a survival phenotype, were exposed to 40% NHS or HIS for 16 h at 34 °C. The cells were plated as a 1% soft agar overlay on BSK-M plates with kanamycin (200 µg/ml) and incubated at 34 °C for 2 weeks; the number of surviving cells was counted and calculated as CFUs^49^.

### Proteinase K accessibility of BOM1093

*B. garinii* HT59G/pBOM1093 was grown ***at 34 °C and collected by centrifugation at 2000×*g* for 15 min at 4 °C and washed twice with PBS containing 5 mM MgCl_2_. The cells (8.3 × 10^7^ cells/tube) were resuspended in 0.5 ml of either PBS or PBS with proteinase K (to a final concentration of 200 µg/ml). All the samples were incubated for 1 h at 20 °C. Reactions were terminated by the addition of Pefabloc SC (Roche Diagnostics, Mannheim, Germany) to a final concentration of 100 mM. Cells were again pelleted by centrifugation (5000×*g* for 10 min at 4 °C), washed twice with PBS containing 5 mM MgCl_2_ and 20 mM Pefabloc SC and resuspended in Laemmli sample buffer (62.5 mM Tris pH6.8, 2% SDS, 10% glycerol, 0.01% bromophenol blue, and 0.5% 2-mercaptoethanol) to carry out sodium dodecyl sulfate–polyacrylamide gel electrophoresis (SDS-PAGE)^56^. The samples corresponding to 1.6 × 10^6^ whole cell equivalents were separated on a 12.5% SDS-PAGE gel, transferred to a Sequi-Blot PVDF membrane (Bio-Rad Laboratories, Hercules, CA, USA), and western blotting was performed as previously described^57^. Monoclonal antibodies (mAb) against borrelial OspA (H5332)^58^, flagellin (H9724)^59^, p100 (Mab958) (Merck, Darmstadt, Germany), or anti-BOM1093 rabbit serum (prepared in this study) were used. To detect these antibodies, Horseradish peroxidase (HRP)-conjugated goat anti-mouse IgG (Jackson ImmunoResearch, West Grove, PA, USA) or rabbit IgG (Merck) were used. HRP-conjugated antibodies were detected by chemiluminescence using the electrogenerated chemiluminescence (ECL) Prime detection reagent (GE Healthcare Bioscience, Piscataway, NJ, USA).

### Pull down assay

*Borrelia garinii* HT59G cells expressed as 6xHis-tagged to *C*-terminal of BOM1093 (10^8^ cells/reaction) were incubated with NHS (20%) for 1 h at 37 °C and washed 3 times with TBS buffer (20 mM Tris–HCl pH 7.0, 0.25 M NaCl) containing 5 mM Pefabloc SC. Hexa-His-tagged BOM1093 obtained from sonicated cells was purified using HisPur Ni–NTA Magnetic Beads (Thermo Fisher Scientific, Waltham, MA, USA).

The sample was separated by 10% SDS-PAGE, transferred to a PVDF membrane, and analyzed using either mAb or polyclonal antibody (pAb) (1:1000) to detect complement regulators for 1 h followed by the HRP-conjugated monoclonal antibody (1:5000) for 1 h. The blot was developed using the ECL Prime detection reagent. For the detection of complement regulators, antibodies were used as follows; Anti-Vn, Anti-clusterin, Anti-FHL-1, Anti-factor I and Anti-complement factor H-related protein 1 mAbs were purchased from R&D Systems (Minneapolis, MN, USA), Anti-C4BP mAb was from Santa Cruz Biotechnology (Dallas, TX, USA), Anti-properdin mAb was from Abcam, Anti-carboxy peptidase N mAb was from Bioss (Woburn, MA, USA), Anti-FH pAb was from Merck, Anti-C1 inhibitor pAb was from Complement Technology (Tyler, TX, USA). To detect these antibodies, HRP-conjugated goat anti-mouse IgG (Jackson ImmunoResearch, West Grove, PA, USA), HRP-conjugated anti-rabbit IgG (Merck) and HRP-conjugated rabbit anti-goat IgG (Abcam) were used.

### Quantitation of binding of Vn to transformants by ELISA

Vn-binding to *B. garinii* HT59G/pBOM1093 was evaluated by ELISA using a 96-well Maxisorp plate (Thermo Fisher Scientific). The wells were coated with 0.5 µg/well of sonicated *B. garinii* HT59G/pBOM1093 or *B. garinii* HT59G/pBSV2 in TBS buffer and the plate was incubated at 4 °C overnight. Next, the plate was washed three times with TBS buffer and treated with blocking buffer (TBS buffer with 1% skim milk) at room temperature for 1 h. After soaking in TBS buffer, the plates were incubated for 2 h at room temperature with recombinant human Vn (Sigma-Aldrich, St. Louis, MO, US) (6.25–400 nM). The plate was then washed 3 times with washing buffer (TBS buffer with 0.05% Tween 20) and incubated with mouse anti-human Vn IgG (1:1000 dilution with blocking buffer) at 37 °C for 1 h. After washing, HRP-conjugated goat anti-mouse IgG (1:5000) was incubated at 37 °C for 1 h. To detect the HRP-labeled secondary antibodies, a 3,3′,5,5′-tetramethylbenzidine solution (TMB; Nakarai Tesque, Kyoto, Japan) was added to the wells and allowed to react for 10 min before the absorbance was measured at 450/620 nm. The values of *B. garinii* HT59G/pBSV2 was subtracted from the values of *B. garinii* HT59G/BOM1093 and the K_D_ was calculated using Prism Ver. 6 (GraphPad Software, San Diago, CA, USA).

### Depletion of Vn from NHS

Vn-depleted human serum (HSΔVn), which retained complement activity, was generated according to the method reported by Hallstrom^60^. A total of 250 µg of protein A/G-sepharose (Abcam) was incubated with 200 µg of anti-Vn polyclonal rabbit serum (Gene Tex, CA, USA) in PBS overnight at 4 °C with mild shaking. Unbound anti-Vn antibody was removed by washing three times with PBS. Thereafter, NHS was added thrice and the mixture was incubated each time for 20 min at 4 °C. The Vn-depleted serum was analyzed for the presence of Vn by ELISA and western blotting. For ELISA, anti-human Vn monoclonal antibody (1:2000) and HRP-conjugated goat anti-mouse IgG monoclonal antibody (1:10,000) were used. The signal was detected using the TMB reagent. For western blotting, the HSΔVn was separated by 10% SDS-PAGE. For detection of Vn, anti-human Vn monoclonal antibody (1:2000) was used. As an internal control, serum clusterin was assessed in this study. Anti-human clusterin monoclonal antibody (1:2000) (Quidel, CA, USA) and HRP-conjugated goat anti-mouse IgG monoclonal antibody (1:10,000) were used to detect serum clusterin. The signal was detected by a chemiluminescence-based technique using ECL Prime detection reagent.

### Evaluation of the effect of Vn depletion activity of NHS

The *B. garinii* HT59G transformants (~ 10^6^ cells/ml) were reacted to HSΔVn with or without 1 mM of recombinant human Vn (Sigma-Aldrich, USA). Briefly, the Vn-depleted serum (the third HSΔVn sample; HSΔVn3^rd^) was incubated in bactericidal assays at a final concentration of 10% (v/v) for 4 h at 34 °C. The cells were plated as a 1% soft agar overlay on BSK-M plates with kanamycin (200 µg/ml) and incubated for 10–14 days at 34 °C.

### Medical ethics

The normal human serum (NHS) was obtained from healthy Japanese blood donors. The blood collection was carried out in accordance with international guideline and regulations (Declaration of Helsinki, 1964). All experimental protocols used human serum, the procedure of blood collection, and documented informed consent were approved by the Ethical Committee of the National Institute of Infectious Diseases for medical research using human subjects (Approval No. 791 on June 26, 2017). All volunteers provided informed consent.

### Statistical analysis

Results were assessed using the Student's t test for paired data. A value of p ≤ 0.01*** was considered statistically significant.

## Supplementary Information

Supplementary Information

## Data Availability

Materials established in this study are available from the corresponding author on reasonable request.
