# Supplementary material for: Vitronectin binding protein, BOM1093, confers serum resistance on Borrelia miyamotoi
Source: Sci Rep. 2021 Mar 9;11:5462. doi: 10.1038/s41598-021-85069-w (PMC7943577; doi:10.1038/s41598-021-85069-w)
Supplement: Supplementary file 1 — Supplementary Information [file 41598_2021_85069_MOESM1_ESM.pdf]

Supplementary information

Vitronectin binding protein, BOM1093, confers serum resistance on *Borrelia miyamotoi*

Kozue Sato[1], Yumi Kumagai[1, 2], Tsuyoshi Sekizuka[3], Makoto Kuroda[3], Tetsuya Hayashi[4], Ai Takano[5], Gaowa[6], Kyle R. Taylor[7], Makoto Ohnishi[1], Hiroki Kawabata[1]*

[1] Department of Bacteriology-I, National Institute of Infectious Disease. Tokyo 162-8640, Japan.
[2] Department of Host Defense and Biochemical Research, School of Medicine, Juntendo University, Tokyo 113-8421, Japan.
[3] Pathogen Genomics Center, National Institute of Infectious Disease. Tokyo 162-8640, Japan.
[4] Department of Bacteriology, Faculty of Medical Sciences, Kyushu University, Fukuoka 819-0395, Japan.
[5] Laboratory of Veterinary Epidemiology, Joint Faculty of Veterinary Medicine, Yamaguchi University, Yamaguchi 753-8511, Japan.
[6] Inner Mongolia Key Laboratory of Tick-borne Zoonotic Infectious Disease, Department of Medicine, College of Hetao, China.
[7] College of Veterinary Medicine, Washington State University, U.S.A.

* Correspondence:
Hiroki Kawabata Ph.D., Department of Bacteriology-I, National Institute of Infectious Disease. Toyama 1-23-1, Shinjuku, Tokyo 162-8640, Japan.
E-mail address: kbata@nih.go.jp
Tel: +81-3-5285-1111, Fax: +81-3-5285-1163

Supplementary Figure S1.

Uncropped Western blots of figure 4

Panel 1B

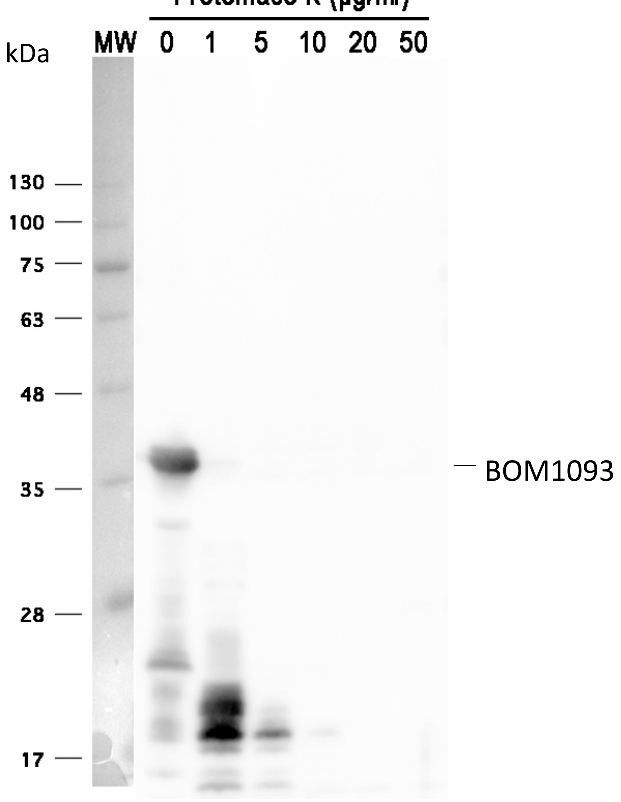

Panel 1C

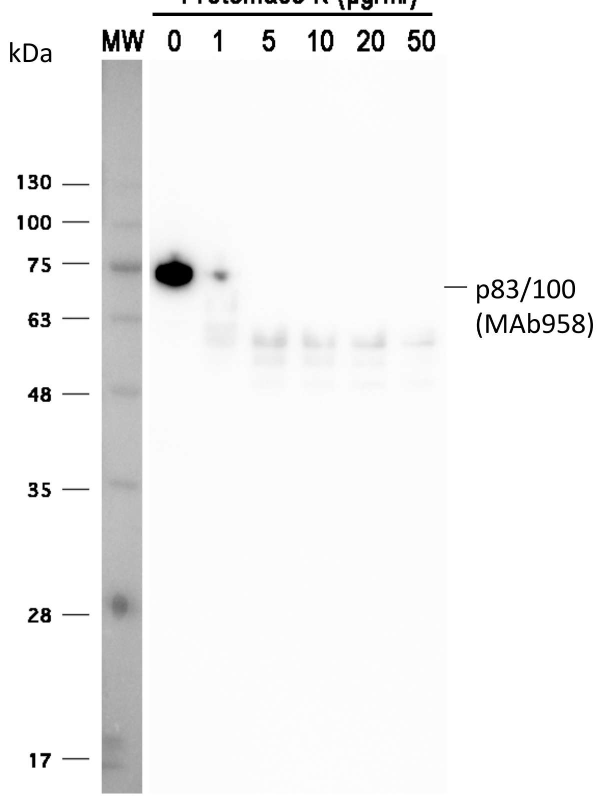

Panel 1D

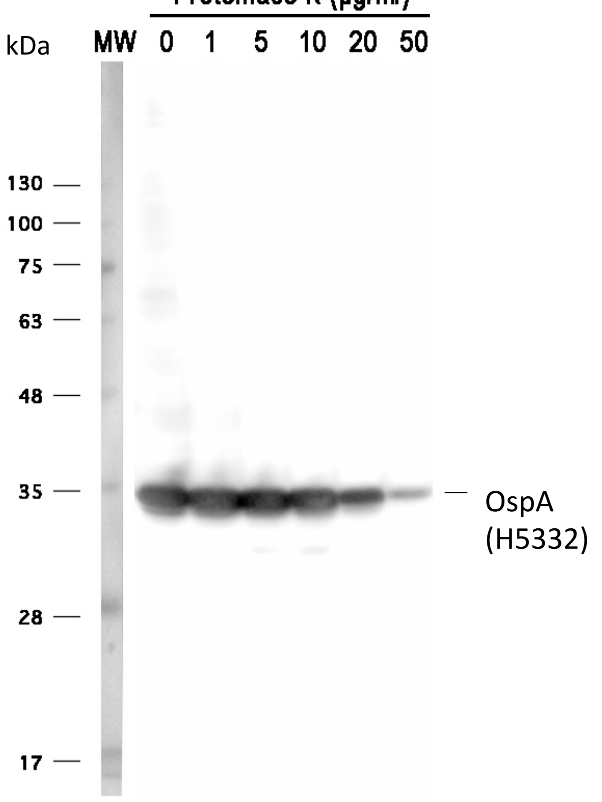

Panel 1E

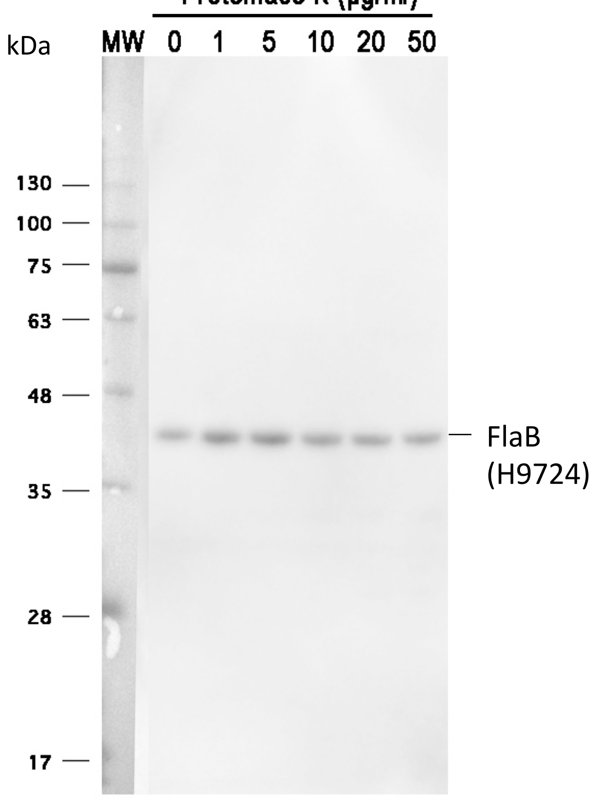

Supplementary Figure S2.

Uncropped Western blots of figure 5

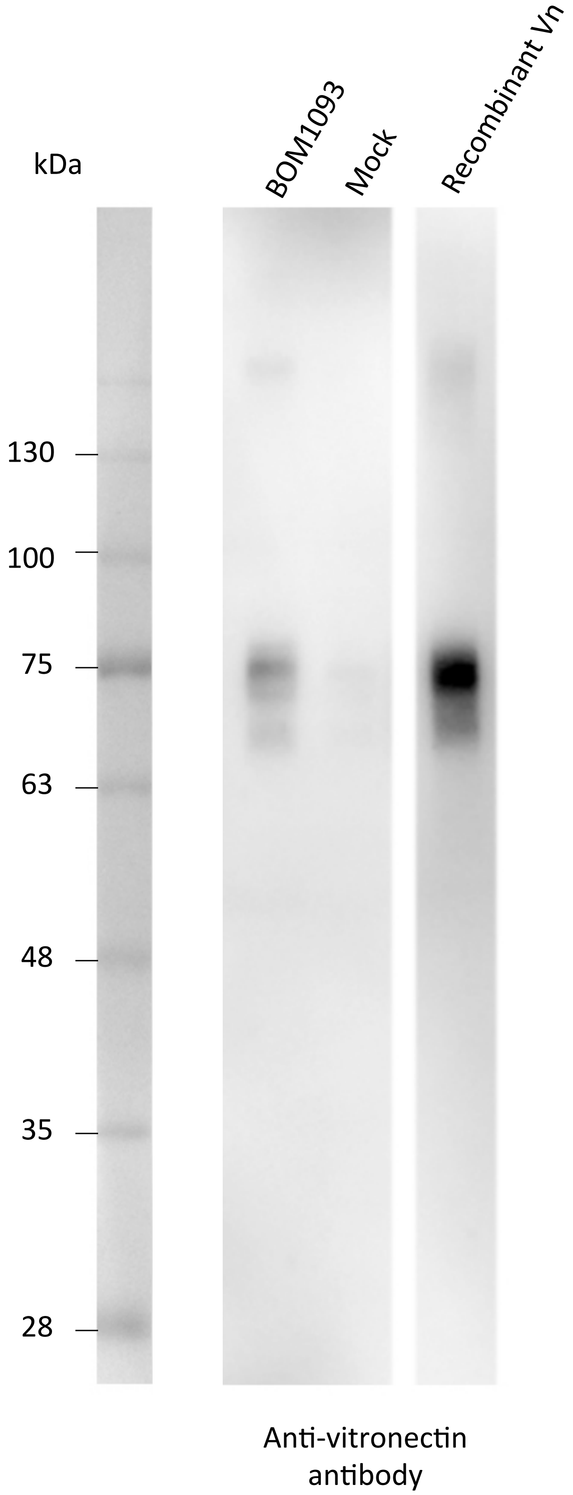

Supplementary Figure S3.

Uncropped Western blots of figure 8, panel A

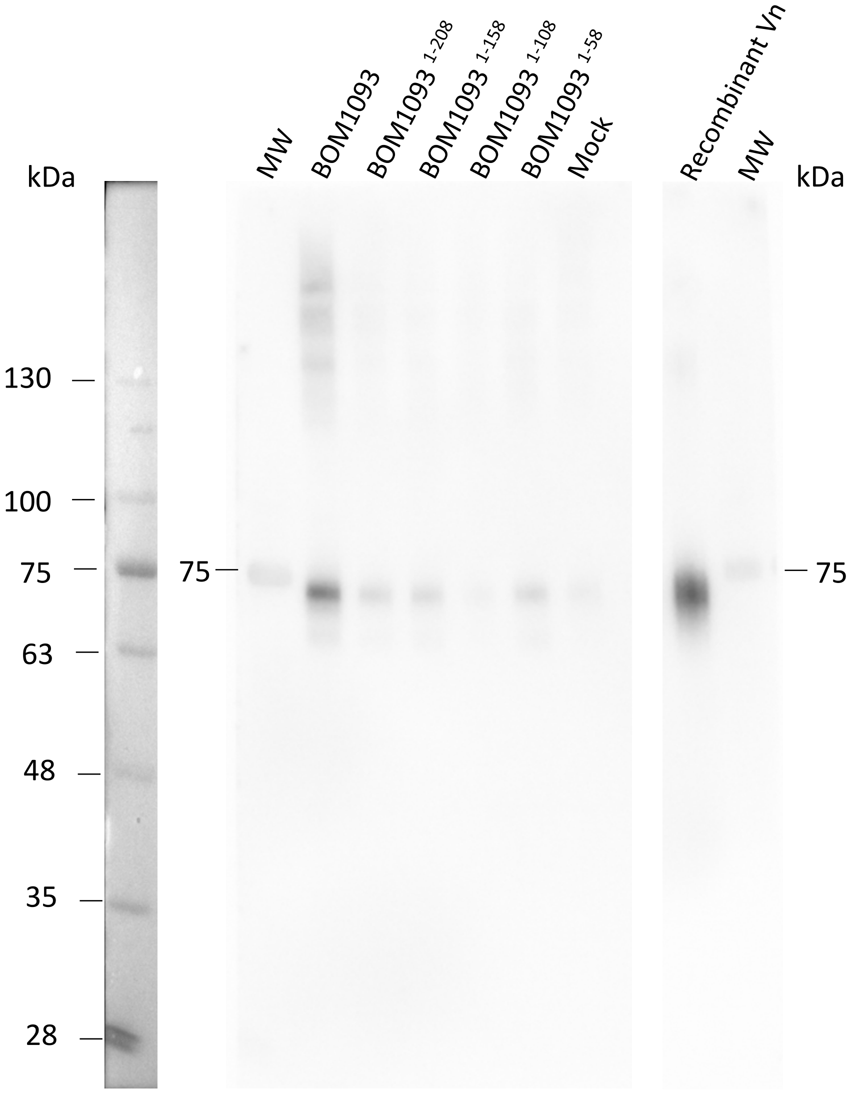

Anti-vitronectin antibody

Supplementary Figure S4.

Uncropped Western blots of figure 9, panel A

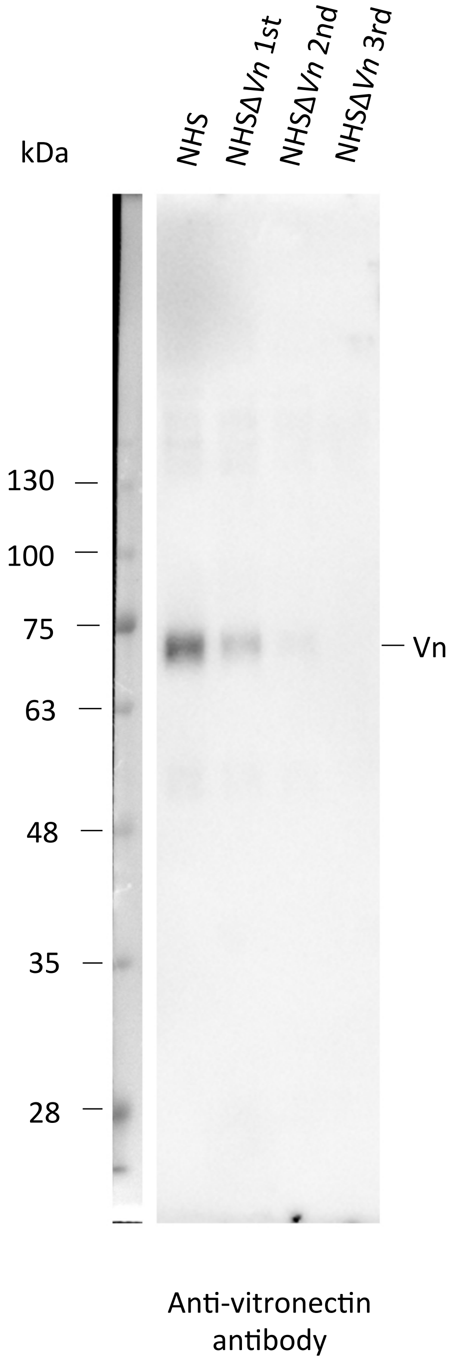

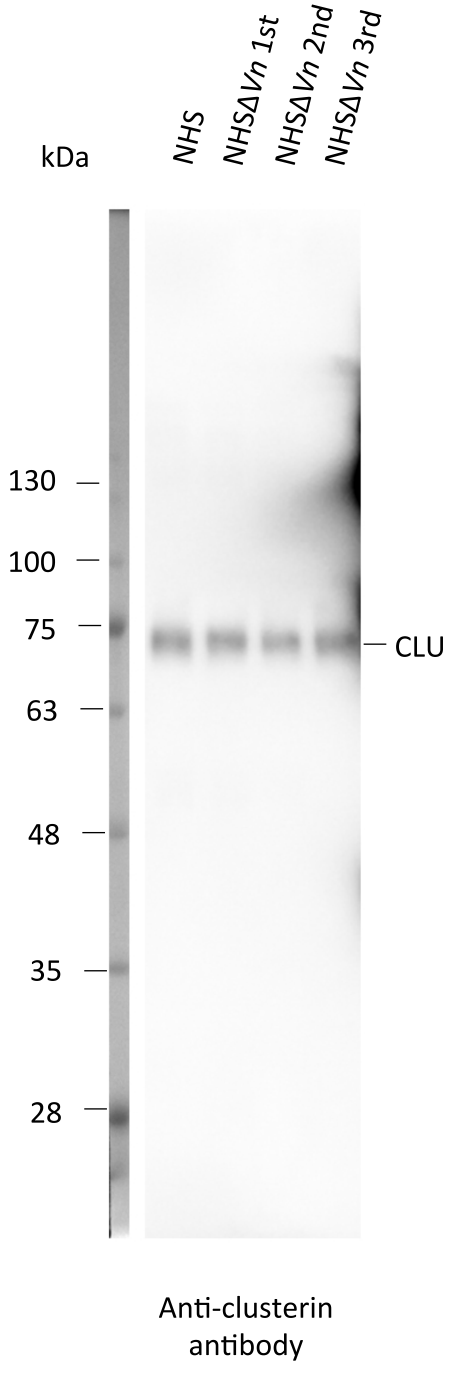

Supplimental table S1. DNA primers used in this study.

Primer name	DNA sequence (5' - 3')	Purpose, in this study
*kanR*_FW	TCAGACTAAACTGGCTGACG	Detection of Kanamycine resisitance gene
*kanR*_RV	AAACTCACCGAGGCAGTTCC	Detection of Kanamycine resisitance gene
T7	TAATACGACTCACTATAGGG	Amplification of multi-cloning site in pBSV2
AS-T	GAGTCCATGTGCTGGCGTTC	Amplification of multi-cloning site in pBSV2
pTM61_pflaB_FW+tag	CCCGGGGATCCTCTAGATAAACTGCCAGGAATTGGGGA	Amplification of *flaB* promotor region encoding on pTM61, InfusionTag+
pTM61_pflaB_RV	ATGTTTTTCCTCCTTATAAAGTTAATCTTT	Amplification of *flaB* promotor region encoding on pTM61
*bom1093*_FW	AAGGAGGAAAAACATATGAAAGGGAGGAGAATATTTATG	Amplification of full length of *bom1093*, InfusionTag+
*bom1093*_RV	GGCCAGTGCCAAGCTTTTACTCAATTGTATCTAAAACTC	Amplification of full length of *bom1093*, InfusionTag+
bom1093 1-208_RV	GGCCAGTGCCAAGCTTTTAAGATTGAAGATCCAATATACT	BOM1093-truncate 209-308, InfusionTag+
bom1093 1-158_RV	GGCCAGTGCCAAGCTTTTACTCCTTATTCTCATCATGCTC	BOM1093-truncate 159-308, InfusionTag+
bom1093 1-108_RV	GGCCAGTGCCAAGCTTTTAAAGCTCTGATATTAATTTTTC	BOM1093-truncate 109-308, InfusionTag+
bom1093 1-58_RV	GGCCAGTGCCAAGCTTTTATTGAACACCATTTACAACCCCTTTTG	BOM1093-truncate 59-308, InfusionTag+
*bom1093*_6XHis_RV	GGCCAGTGCCAAGCTTTTAATGATGATGATGATGATGCTCAATTGTATCTAAAACTC	BOM1093-non truncate, InfusionTag+6XHis
bom1093 1-208_6XHis_RV	GGCCAGTGCCAAGCTTTTAATGATGATGATGATGATGAGATTGAAGATCCAATATACT	BOM1093-truncate 209-308, InfusionTag+6XHis
bom1093 1-158_6XHis_RV	GGCCAGTGCCAAGCTTTTAATGATGATGATGATGATGCTCCTTATTCTCATCATGCTC	BOM1093-truncate 159-308, InfusionTag+6XHis
bom1093 1-108_6XHis_RV	GGCCAGTGCCAAGCTTTTAATGATGATGATGATGATGAAGCTCTGATATTAATTTTTC	BOM1093-truncate 109-308, InfusionTag+6XHis
bom1093 1-58_6XHis_RV	GGCCAGTGCCAAGCTTTTAATGATGATGATGATGATGTTGAACACCATTTACAACCCCTTTTG	BOM1093-truncate 59-308, InfusionTag+6XHis

Infusion Tags and 6xHis Tag were designated by red and blue litereture, respectively.

Supplimental table S2. DNA primers used for amplification of target ORFs with in-fusion tag in this study.

	DNA sequence (5' - 3')	
Amplification target	Forward	Reverse
bom879	AAGGAGGAAAAACATATGAAAGAAATAGGCATATCAAC	GGCCAGTGCCAAGCTTTTATTGATGCTCTATAATCTTA
bom883	AAGGAGGAAAAACATATGAAAAATTACATTTTTATCTTAC	GGCCAGTGCCAAGCTTCTAATTTACTACTTTTAGAAT
bom885	AAGGAGGAAAAACATATGATTTTTTGCTATAATTGTATTG	GGCCAGTGCCAAGCTTTCATAAATATCTTGTATTTATAAC
bom886	AAGGAGGAAAAACATATGAAGTATAATATTTTTGTATTACTAGC	GGCCAGTGCCAAGCTTTCAATTATCGTTATAGTCTAATTTG
bom887	AAGGAGGAAAAACATATGAAAAAAAATATTTTAAACAACAC	GGCCAGTGCCAAGCTTTTATTTTATTGTATCTAGAATTG
bom888	AAGGAGGAAAAACATATGAGAAAGAATATTTTAAATAACGC	GGCCAGTGCCAAGCTTTTATATTTTAGATAAATTATTTGTAAC
bom897	AAGGAGGAAAAACATATGAAAGAGTTTGGTATGGTATTATTG	GGCCAGTGCCAAGCTTCTATATAACGCTACTAATTAAATTG
bom899	AAGGAGGAAAAACATATGAAGAGTTTGAATTATAATTTTATTTG	GGCCAGTGCCAAGCTTCTAAGGACTCAAATAAGCATATA
bom900	AAGGAGGAAAAACATATGAGAAAGTTTTATATAGTGTTTTGTG	GGCCAGTGCCAAGCTTTTACAATATCTTAAGTAAACTATTAAC
bom902	AAGGAGGAAAAACATATGCAAATTAGAGTTATATTTTATATTTTA	GGCCAGTGCCAAGCTTTCATATCCAAGGATTGCATAAATG
bom907	AAGGAGGAAAAACATATGGAGAGAAGTAAAATGCAAAAAAT	GGCCAGTGCCAAGCTTTTAGTTGTTAATAGGAACTCTG
bom909	AAGGAGGAAAAACATATGAAGAGGATTAATTTTAATTTTGTTTTG	GGCCAGTGCCAAGCTTTCAAGTATAAATTTTTGTATCATCTG
bom910	AAGGAGGAAAAACATATGGTTAAATTTATAAATTATTTGATAC	GGCCAGTGCCAAGCTTTTACTTTTCCGAAAGTTCCTTT
bom929	AAGGAGGAAAAACATATGTTTATTAAATTTACCATGATTGT	GGCCAGTGCCAAGCTTCTAAAAATCATATTTCTTTATAAA
bom932	AAGGAGGAAAAACATATGAGGAGAGATATGAAAAATTTCAG	GGCCAGTGCCAAGCTTTTAATCTTCTTCTTCAAAATCTC
bom935	AAGGAGGAAAAACATATGTTAAGGATTTTGATGTTAAGTTT	GGCCAGTGCCAAGCTTTCAAGAGCTTATTAAATTATCAA
bom936	AAGGAGGAAAAACATATGAAAAATTTACTATTAATAATACC	GGCCAGTGCCAAGCTTTTAAAGTTTAAAATCAGGTGC
bom937	AAGGAGGAAAAACATATGAAAAAAATCATTCTAAGTGGTATG	GGCCAGTGCCAAGCTTTTATGCTGATAGACTATCTCG
bom945	AAGGAGGAAAAACATATGAAAAAAAAATTAACTCAAAGATTAC	GGCCAGTGCCAAGCTTTTACTTTTTAAATTTACTATATTTAT
bom962	AAGGAGGAAAAACATATGAAAAAAATTATTCATTTACTTC	GGCCAGTGCCAAGCTTTTAATTTTTTAGTTTTGAATACC
bom975	AAGGAGGAAAAACATATGAGAAGAATTAATGTTGTTTTG	GGCCAGTGCCAAGCTTTTAACCACCTGATCCTGC
bom1002	AAGGAGGAAAAACATATGAATAAAAATATAAATTATTTAATAATAC	GGCCAGTGCCAAGCTTTTAGCAACCACTTTTGACTTTC
bom1036	AAGGAGGAAAAACATATGGTCAAATTTATATATTATTTAATGC	GGCCAGTGCCAAGCTTTTAACAACCACTTGTCACTTTG
bom1063	AAGGAGGAAAAACATATGTTTTTAAGTTTATTGCTATTTATTAC	GGCCAGTGCCAAGCTTTTAAATAATTCCAAACACAAATCC
bom1072	AAGGAGGAAAAACATATGAGACATAAGTATATTGACATTG	GGCCAGTGCCAAGCTTCTAATTACCCTTCTCAGGAA
bom1092	AAGGAGGAAAAACATATGATTTTTAAAAATAAATATTTAGC	GGCCAGTGCCAAGCTTTTATCTTGCAACAATACCTG
bom1095	AAGGAGGAAAAACATATGAAGAAGAGTATTTTGGCAGTATG	GGCCAGTGCCAAGCTTTTATCTTTCAAGATTCTCTAGAAC
bom1103	AAGGAGGAAAAACATATGGAGAAAAGAAGCGAGAGAAAG	GGCCAGTGCCAAGCTTTTACTCTGCTGTTTTTGAGTTTC
bom1109	AAGGAGGAAAAACATATGAGTAAAAGAAAAACATTAAGTGC	GGCCAGTGCCAAGCTTTTATTGCTGTGCGCTAGCTG
bom1110	AAGGAGGAAAAACATATGAGTAAAAGAAAAACATTAAGTGC	GGCCAGTGCCAAGCTTTTACTTCTTTACACTAGCTGT
bom1125	AAGGAGGAAAAACATATGAATTATTATATGCGCATATTTTTGG	GGCCAGTGCCAAGCTTTCACTTAGCAAAGTATTTAAGA
bom1127	AAGGAGGAAAAACATATGAGGTATACTTTTTTCTCATTTGTC	GGCCAGTGCCAAGCTTCTATAAAATATTATTTTCAAGAGC
bom1134	AAGGAGGAAAAACATATGAAAATTCTAAAAAACACTTTTATG	GGCCAGTGCCAAGCTTTCATAATCTTCCTTCCTTAA
bom1137	AAGGAGGAAAAACATATGTTATCTAGGAGTATTATGATAG	GGCCAGTGCCAAGCTTTCAAAGTGAGGAAGGAG
bom1167	AAGGAGGAAAAACATATGAATAAAAATATAAGTTATTA	GGCCAGTGCCAAGCTTTTATTGGCAACCACTTTTG
bom1198	AAGGAGGAAAAACATATGAGTTATTATTACAATGC	GGCCAGTGCCAAGCTTTTATATTATATGCACATTTT
bom1218	AAGGAGGAAAAACATATGTTTATGGTTAAATTTATAT	GGCCAGTGCCAAGCTTTTAACAACTACTTGTCACTTG
bom1226	AAGGAGGAAAAACATATGGTTAAATTTATATATTATTT	GGCCAGTGCCAAGCTTTTATAACCAAGTTGTTCTA
bom1273	AAGGAGGAAAAACATATGAATAAGTTATTTGTAGAT	GGCCAGTGCCAAGCTTCTACTCCTTTAAGGTCAC
bom1280	AAGGAGGAAAAACATATGATAAATAATGTAATTATATT	GGCCAGTGCCAAGCTTAAAGTTTGAATAATTGGAG
bom1283	AAGGAGGAAAAACATATGAACTGTATAAATAATTTATTAGATC	GGCCAGTGCCAAGCTTTTAACTTTGAGTTTCTAAAAG
bom1318	AAGGAGGAAAAACATATGAAAAATAATATTTTAAAAAAC	GGCCAGTGCCAAGCTTTTATTGGTTGTCTATTAAAAT
bom1319	AAGGAGGAAAAACATATGAAAAGAATTAACTTTATTC	GGCCAGTGCCAAGCTTTTAACTTCCTACTGCATCG

bom1320	AAGGAGGAAAAACATATGATAATATCTGTAAATCAT	GGCCAGTGCCAAGCTTTTATAAGTCATTTCTCCAAT
bom1322	AAGGAGGAAAAACATATGAGAAGAATTAATTTTGTT	GGCCAGTGCCAAGCTTTTAACCACCTACTCCAC
bom1323	AAGGAGGAAAAACATATGATAAGATATATAAATTATTT	GGCCAGTGCCAAGCTTTTAATTAGCATTTCCACATT
bom1333	AAGGAGGAAAAACATATGAAAAAAATTATTCATTTATT	GGCCAGTGCCAAGCTTTTAATTTTTTAGTTTTGAATAC
bom1349	AAGGAGGAAAAACATATGAGTAAAAGAAAAACATTAAC	GGCCAGTGCCAAGCTTAGCATTTCCTACACCTTTAAG
bom1350	AAGGAGGAAAAACATATGAGTAAAAGAAAAACATTA	GGCCAGTGCCAAGCTTCTAACTTGTTGATTCTGTTG
bom1351	AAGGAGGAAAAACATATGAGTAAAAGAAAAACATTAAG	GGCCAGTGCCAAGCTTTTATTGCTGTGCGCTAGCT
bom1352	AAGGAGGAAAAACATATGAAAAAAAGAAAAACATTAAG	GGCCAGTGCCAAGCTTTTAGCTTTTTACATTAGTTCA
bom1355	AAGGAGGAAAAACATATGACATTATTTTTAGGATTAG	GGCCAGTGCCAAGCTTTTATACTATATCTTGTACTTTC
bom1357	AAGGAGGAAAAACATATGTATATAAATAAGGATGC	GGCCAGTGCCAAGCTTTTAATTCTCACCACCAGC
bom1358	AAGGAGGAAAAACATATGAAAAAAAGAAAAACATTAAG	GGCCAGTGCCAAGCTTTTAGTTTTTTACACTAGCTG
bom1363	AAGGAGGAAAAACATATGAAAAAAATGAGAAAAAGAAAAAA	GGCCAGTGCCAAGCTTTTATCTTATCCCTGATTGATCC
bom1364	AAGGAGGAAAAACATATGACTTTATTTTTAGGATTAGTG	GGCCAGTGCCAAGCTTTTATTTACTTTTAGCTTCAGAGG
bom1385	AAGGAGGAAAAACATATGAACATAAGTAAAAAATTAAAAT	GGCCAGTGCCAAGCTTCTACCTCTTAGCTATATCTC
bom1399	AAGGAGGAAAAACATATGTTTAAAACACTAATATTAACC	GGCCAGTGCCAAGCTTTTAAATGTTAGACTCGATTATATG
bom1423	AAGGAGGAAAAACATATGAAAAGAAAAACATTAAGTG	GGCCAGTGCCAAGCTTTTACTTACTTTTAACTTCAGACG
bom1429	AAGGAGGAAAAACATATGGAGAAAAGAAGCGAGAGA	GGCCAGTGCCAAGCTTTTACTCTGCTGCTTTTGGATTTC
bom1430	AAGGAGGAAAAACATATGACTTTATTTTTAGGATTAGTG	GGCCAGTGCCAAGCTTTTATTTACTCGTAACTTCAGATAT
bom1435	AAGGAGGAAAAACATATGAAAAAAAGAAAAACATTAAGTG	GGCCAGTGCCAAGCTTTTAGTTGCTCACACTACTAGC
bom1439	AAGGAGGAAAAACATATGACTTTATTTTTAATAATAAATATTGT	GGCCAGTGCCAAGCTTTTAACCAGAAGTTGCTGCTTT
bom1440	AAGGAGGAAAAACATATGAATAGGGTAATAAGTGTATC	GGCCAGTGCCAAGCTTCTATCTATCCATTATCATTCTTAT
bom1441	AAGGAGGAAAAACATATGAATTATTATTACAATGCTTTTA	GGCCAGTGCCAAGCTTTTATTCAATAATATTAATGAACGTA
bom1467	AAGGAGGAAAAACATATGAAAAAAAGAGAAACATTAAGTGAG	GGCCAGTGCCAAGCTTTTATGATTGACCAATAGTTGCT
bom1468	AAGGAGGAAAAACATATGAATAAGAGTGTTTTGGCATT	GGCCAGTGCCAAGCTTCTATTTCTTTCTTTCCATTGC
bom1470	AAGGAGGAAAAACATATGATTAGGTTATTTAAAGTATTG	GGCCAGTGCCAAGCTTCTATCTTTTACTAACTCCTAATAC
bom1487	AAGGAGGAAAAACATATGACTTTATTTTTAATAATAAATATT	GGCCAGTGCCAAGCTTTTAACTTCCACTAACAGCAGG
bom1489	AAGGAGGAAAAACATATGAGTAAAAGAAAAACATTAAGTG	GGCCAGTGCCAAGCTTTTACTTCTGTGCACTAGTTGTT
bom1490	AAGGAGGAAAAACATATGAGTAAAAGAAAAACATTAAGTG	GGCCAGTGCCAAGCTTTTATAATTCTTTATTTTTTACACTAG
bom1493	AAGGAGGAAAAACATATGATATCTTGTGGAAATGGGG	GGCCAGTGCCAAGCTTTTAACCAGAAGTTGCTGCTT
bom1515	AAGGAGGAAAAACATATGAAAGGGAGGAGAATATTTATG	GGCCAGTGCCAAGCTTTTACTCAATTGTATTTAGAACAC
bom1519	AAGGAGGAAAAACATATGATTTTGGCAGTATGTATATTAATAT	GGCCAGTGCCAAGCTTTTATCTTTCAAGATTCTCTAGAA
cspZ (B31)	AAGGAGGAAAAACATATGAAAAAAAGTTTTTTATCAATATACATG	GGCCAGTGCCAAGCTTCTATAATAAAGTTTGCTTACTAG

Infusion Tags was designated by red literetured, respectively.
